# Design of a personalized oral‒motor exercise device for speech impairment rehabilitation

**DOI:** 10.3389/fbioe.2025.1543259

**Published:** 2025-03-27

**Authors:** Seong Tak Woo, Ji-Wan Ha, Sungdae Na

**Affiliations:** ^1^ Department of Electronic Engineering, Dong Seoul University, Seongnam, Republic of Korea; ^2^ Department of Speech and Language Pathology, Daegu University, Gyeongsan, Republic of Korea; ^3^ Department of Biomedical Engineering, Kyungpook National University Hospital, Daegu, Republic of Korea

**Keywords:** dysarthria rehabilitation, oral-motor exercise, self-calibration, speech therapy, tongue movement

## Abstract

**Introduction:** Dysarthria is a speech disorder that stems from impaired muscle control due to lesions in the articulatory system, necessitating targeted rehabilitation exercises to strengthen affected muscles. Current devices used for rehabilitation often fail to accurately assess exercise execution, which limits their effectiveness.

**Methods:** This study introduces a novel oral-motor rehabilitation device designed to overcome these limitations. The device features flexible sensors and a signal processing module that provides real-time feedback on training intensity. It is integrated with a mobile application that enables users to monitor their tongue’s range of motion and track their progress through a calibration process that uses a simple moving average filter. A preliminary study was conducted with five healthy adult male subjects to verify the device’s basic operational characteristics.

**Results:** The effectiveness of the device in improving muscle function and regulating training intensity was evaluated using the Iowa Oral Performance Instrument. The results showed promising outcomes in enhancing articulation and oral-motor skills, indicating that the device could effectively contribute to dysarthria rehabilitation.

**Discussion:** By addressing the gaps in current rehabilitation practices for dysarthria, the proposed device offers a comprehensive and personalized approach to oral-motor therapy. Its ability to provide immediate feedback and track progress can significantly enhance the rehabilitation process, potentially leading to better outcomes for patients with dysarthria.

## 1 Introduction

An articulation disorder or dysarthria are a speech sound disorder characterized by difficulty in accurately producing specific sounds or speech patterns. This results in mispronunciations, substitutions, omissions, or distortions of sounds, often hindering clear communication and making speech difficult to understand (Sugden et al., 2016; Lof, 2003; Sharp and Hillenbrand, 2008). While common during early language development in young children, articulation disorders that persist beyond the expected age typically require speech therapy (Kollia et al., 2019). The causes of this disorder can include developmental delays, physical impairments, neurological conditions, or environmental factors. To diagnose dysarthria, speech-language pathologists assess sound production and muscle coordination, suggesting treatments focused on speech therapy and oral‒motor exercises. Early intervention plays a crucial role in achieving successful communication outcomes (Landa, 2007). Dysarthria is a common consequence of stroke, typically resulting from lesions in various brain regions. About half of all stroke patients experience dysarthria during the acute phase of their stroke. Geddes JM et al. (Geddes et al., 1996) reported that after the acute phase, the prevalence of residual impairments decreases to 27% in the following 6 months. These communication disorders associated with dysarthria significantly impact the quality of life, highlighting the need for a standardized protocol to measure speech impairments and predict outcomes effectively. According to a literature review by Chiaramonte R and Vecchio M (Chiaramonte and Vecchio, 2021), several methods are mentioned to determine the severity of dysarthria and the effectiveness of rehabilitation treatment. In general, there is the Frenchay Dysarthria Assessment-2 (FDA-2) for the diagnosis and follow-up of dysarthria, and this assessment tool is used to observe the severity by evaluating the muscle functions around the oral cavity, such as breathing, oral movement, and vocal cord function (Hijikata et al., 2022). Additionally, acoustic analysis software like MDVP (Kent et al., 2003) and Praat (Kim and Jo, 2013) are utilized to objectively measure speech’s acoustic parameters, providing indices that reflect articulatory movements. These programs are particularly useful for tracking changes in the formant frequencies F1 and F2 following speech therapy, offering insights into the characteristics of articulatory movements by analysing the vocal tract’s shape (Mou et al., 2018).

Unlike voice analysis programs and assessment tools such as MDVP, Praat, or the FDA-2, key devices specifically designed for oral-motor rehabilitation include the Abilex device (Cunningham, 2020) and the tongueometer (Curtis et al., 2023). These tools are essential in speech therapy and oral rehabilitation, especially for individuals recovering from neurological conditions. The Abilex device enhances speech, swallowing, and oral–motor skills by targeting the muscles responsible for articulation and motor coordination. Its ergonomic design and adjustable resistance make it a portable, user-friendly solution for gradual muscle strengthening and personalized therapy (Izumi and Akifusa, 2021; Kamarunas et al., 2024). Meanwhile, the tongueometer measures tongue strength, movement, and endurance, providing clinicians with objective data to evaluate and train oral muscle function. Its real-time feedback and customizable resistance enhance its effectiveness in improving speech clarity and swallowing efficiency (Curtis et al., 2023; Izumi and Akifusa, 2021; Kamarunas et al., 2024; Drulia et al., 2024). In this study, we introduce a new type of oral motor exerciser designed to improve upon the limitations of current oral rehabilitation devices. Unlike the Abilex device, which lacks a sensor and signal processing unit, making feedback on patient oral movements challenging, our device incorporates a resistance flexible film sensor. This allows for linear output characteristics that directly correspond to the range of tongue movement. Additionally, compared to the tongueometer, which uses an airflow tube sensor and thus struggles to provide linear pressure characteristics, our device ensures more accurate and responsive feedback according to the tongue’s movement. Thus, this study aims to develop an oral–motor exerciser tailored to individual tongue strength and to evaluate the range of tongue motion along on its trajectory.

## 2 Method

### 2.1 Implementation of the oral-motor exerciser and user feedback application

**FIGURE 1:**
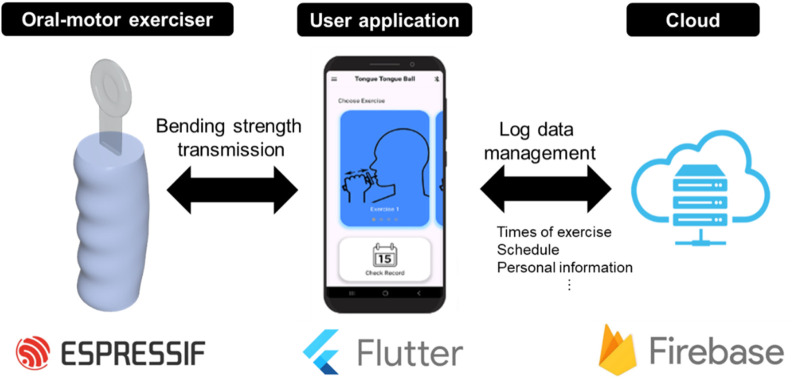
Schematic of the proposed oral‒motor exerciser. (left) The device consists of a flexible sensor, a signal processing unit, and a user-friendly case. The device is connected to the user’s mobile phone or monitoring device (median) to provide real-time data communication and GUI information (implemented with the Flutter program) wirelessly. In addition, it is implemented to provide service functions such as storage of user log data and schedule management by connecting to a mobile phone through the (right) Cloud platform (implemented with the Firebase program).

The conceptual diagram of the proposed oral–motor exerciser is presented in Figure 1.

The exerciser was fabricated using a resistance-based flexible film and an ESP-IC-based signal processing module. Additionally, a dedicated user application was developed to display numerical values for training levels and exercise performance. The oral–motor exerciser was designed to maintain a specific range of motion during training. To this end, a user-customized calibration function was implemented, allowing reference points to be set by measuring the tongue’s range of motion and movement intensity prior to exercise. To evaluate the performance of the oral–motor exerciser, tongue pressure during movement was measured using the Iowa Oral Performance Instrument (IOPI), a standard device for quantifying tongue pressure (Oh, 2022). The IOPI is widely used to measure the maximum strength and endurance of the tongue, as well as the maximum strength of the lips (Adams et al., 2013). It serves as an effective tool for quantitatively evaluating the muscles associated with articulatory functions in the oral cavity (Pitts et al., 2022). This makes it particularly valuable for diagnosing speech and swallowing disorders (Park et al., 2015).

**FIGURE 2:**
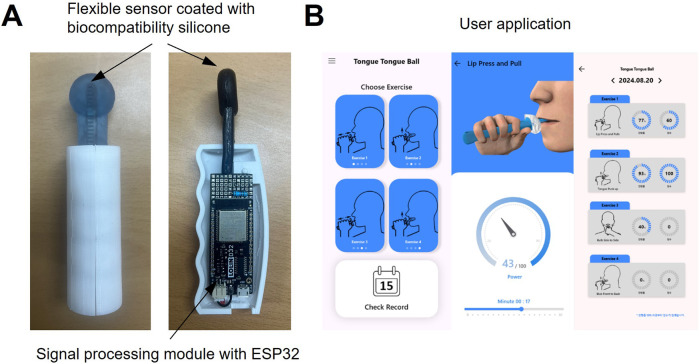
**(A)** Photograph of the fabricated oral‒motor exerciser and **(B)** mobile application for users.

The oral–motor exerciser and its accompanying user feedback application are illustrated in Figure 2. As depicted in Figure 2A, the sensor component of the device is fabricated using a flexible resistive film coated with biocompatible silicone (Sylgard-184, Dow Corning, USA). The flexible film exhibits a variable resistance of 10 kΩ (±3 kΩ), depending on the degree of bending. This feature allows indirect measurements of the bending angle and the force exerted by external pressure. Additionally, the device incorporates a signal processing board with an ESP32 (Espressif Systems, China) IC, enabling wireless functionality for user convenience. As depicted in Figure 2B, a dedicated mobile application is also developed to guide users in their training. The application communicates with the exerciser device via Bluetooth low energy communication and tracks exercise records, encouraging users to build consistent exercise habits.

On the application’s home screen, users can select one of two exercises—tongue push-up or tongue side-to-side movements. Visual demonstrations of the chosen exercise are displayed to help users follow along accurately. The range of motion for each exercise is represented as a gauge, motivating users to maintain their movements within a specific range. All exercise records are stored on a web-based server (Firebase), allowing users to access their progress in real-time. This feature further supports the development of regular exercise routines.

**FIGURE 3:**
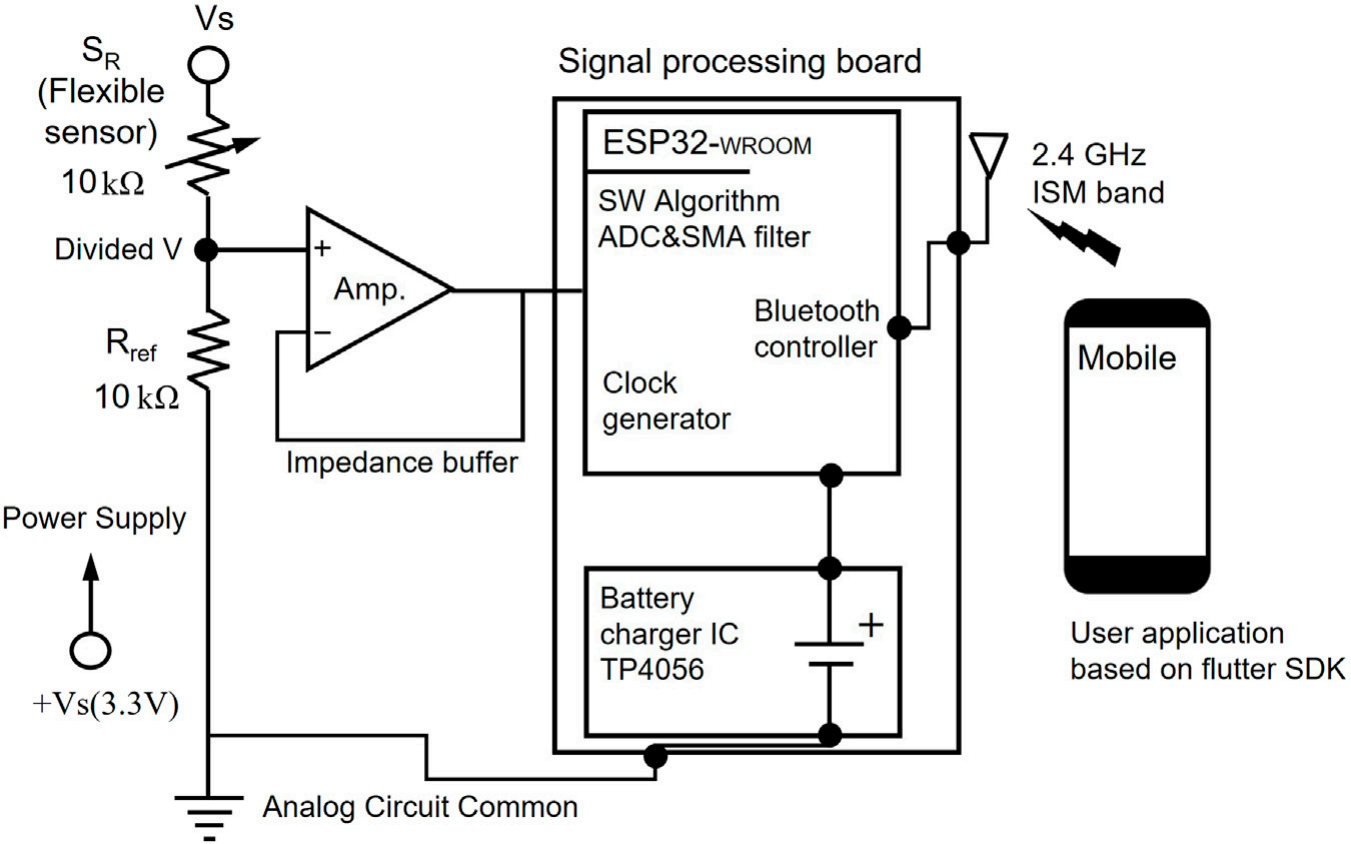
Schematic of the proposed circuit designed for acquiring tongue movement data.

The circuit diagram in Figure 3 depicts the design of a sensor-based data acquisition and communication system equipped with an ESP32 microcontroller for signal processing and wireless data transmission.

The external sensing component comprises a flexible sensor and a resistive element that adjusts its resistance in response to external stimuli, specifically bending during tongue exercises. To ensure accurate signal acquisition, the system incorporates an impedance buffer using an operational amplifier. This buffer minimizes loading effects caused by the high impedance of the flexible sensor. The ESP32 microcontroller serves as the core of the signal processing system, integrating multiple components for efficient data handling and communication. The analog-to-digital converter of the microcontroller digitizes the buffered analog signals, enabling further digital processing and analysis. A simple moving average (SMA) filter is applied during calibration to reduce noise and fluctuations by averaging successive samples, producing a more stable and consistent output. The signal processing board also features an embedded Bluetooth controller operating on the 2.4 GHz ISM band, enabling real-time, wireless data transmission to external devices. This functionality allows seamless communication with a mobile application developed using the Flutter SDK.

### 2.2 SMA filter for self-calibration

Conventional tongue pressure meters measure the absolute pressure generated during tongue‒palate contact (Iskarous, 2005; Green and Wang, 2003; Mefferd, 2016). However, individual differences in the range of tongue motion and contact pressure require a calibration function to ensure accurate and personalized device use. To address this, a calibration function based on the SMA filter was implemented in this study, enabling individualized training. The SMA filter is widely used in biosignal processing owing to its simplicity and effectiveness in reducing noise. Biosignals, such as electrocardiograms (Chen and Chen, 2003), photoplethysmography (Lee et al., 2007), and electromyograms (Hidayat et al., 2015), often contain noise from electrical interference or muscle artifacts, which can obscure critical signal features and complicate analysis. The SMA filter smooths out the signal, making trends and patterns more discernible. It operates by averaging a fixed number of consecutive data points, referred to as the window size. Each data point is replaced with this calculated average, and the window incrementally shifts across the signal, recalculating the average at each step. This process produces a smoothed signal, reducing fluctuations and enhancing signal clarity. In this study, the SMA filter was employed to create a personalized calibration function for the tongue pressure meter. This approach was aimed at improving the device’s ability to account for individual differences in tongue performance during training.

The mathematical representation of the SMA filter used for calibration is as detailed in Equations 1–3. In these equations, *N* denotes the window size*,* $x_{i}$ represents the *i*th data point, and ${avg}_{k}$ signifies the average value within the *k*th window.

$${avg}_{k}=\frac{1}{N}\;\sum_{i=0}^{N-1}{\;x}^{i}$$ (1)

Furthermore, as indicated in Equation 2, the derived average value is weighted by combining the average of the data in the current *k*th window with the average of the data in the subsequent *k* + 1st window, shifted by a single sample point.

$${avg}_{k\;}=\alpha \cdot {avg}_{current}+\left(1-\alpha \right)\cdot {avg}_{k+1}$$ (2)

Here, ${\text{avg}}_{\text{current}\;}$ denotes the average of the data in the current window, ${\text{avg}}_{k+1}$ represents the average of the newly updated data in the next window, and α signifies the weighting factor, defined as

$$\alpha =\frac{WindowSize-MovingPoint}{WindowSize}$$ (3)

During the calibration process, data obtained from the oral–motor exerciser were sampled at a rate of 10 Hz, with 150 samples recorded during both contraction and relaxation phases. To determine optimal parameters, window sizes of 10, 20, 30, 40, and 50 samples were tested, along with moving points set at 10%, 20%, and 50% of the window size. The performance of each configuration was evaluated by measuring the standard deviation of the resulting averages.

**FIGURE 4:**
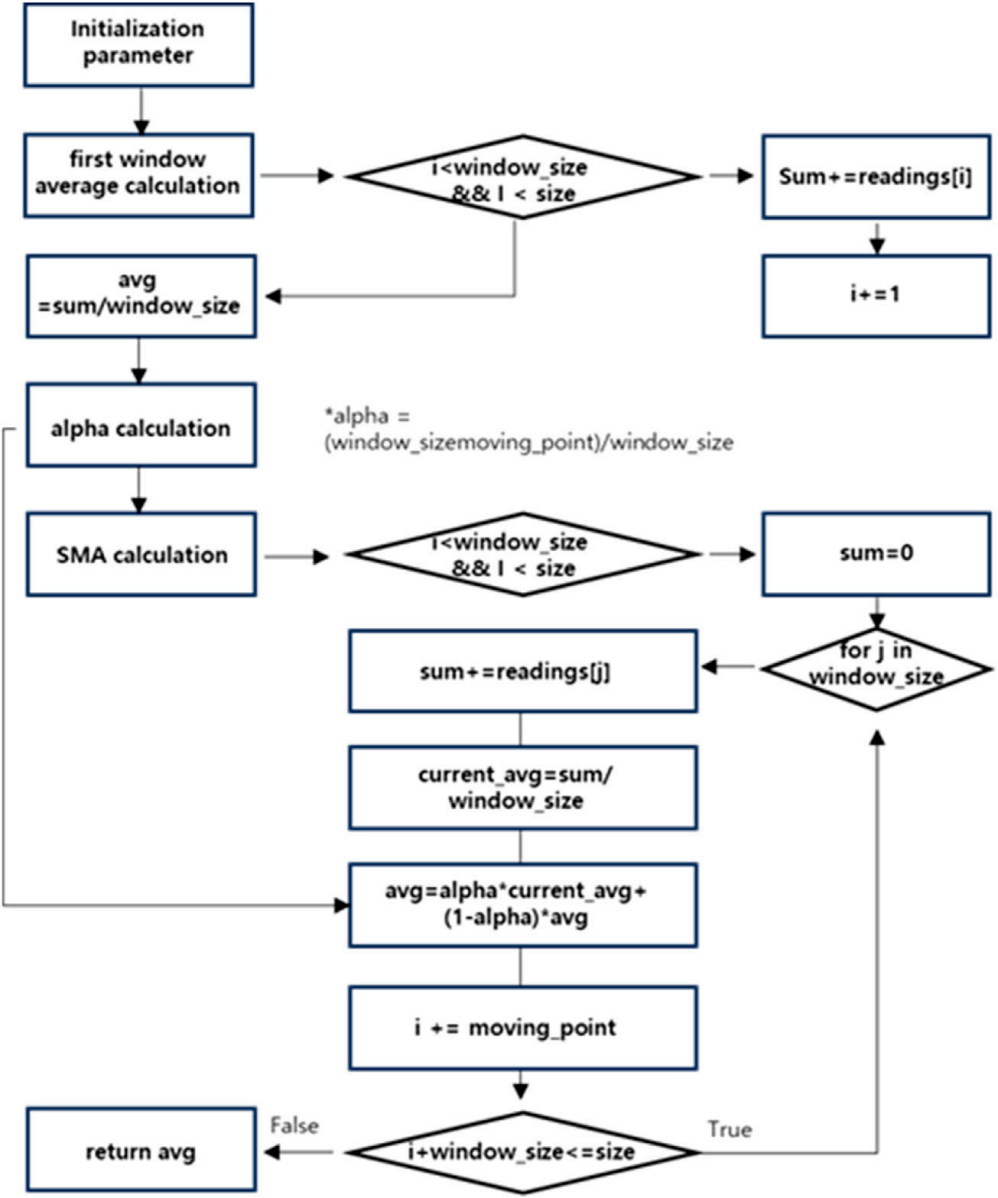
Flowchart of the proposed SMA filter algorithm.

Table 1 presents the pseudocode of the proposed algorithm, and Figure 4 presents the corresponding flowchart. In signal processing, especially when working with sensor data, managing noise is critical to ensure stable outputs. The pseudocode outlines a method for calculating a smoothed average of sensor readings using a sliding window and exponential smoothing.

**Table 1:** Pseudo code for the proposed method based on the SMA filter.

Moving Average Filter	
1	Data = analogRead (flexPin)
2	sumValue = sum (reading [:window_size])
3	Avg [init] = sumValue/window_size
4	
5	alpha = (window_size–moving_point)/window_size
6	
7	for i in range (start_point, len (reading)–window_size+1, moving_point)
8	current_avg = sum (reading [i: i + window_size])/window_size
9	avg = alpha*avg + (1–alpha)*current_avg
10	
11	Return avg

The algorithm begins by reading sensor data from an analog input pin and calculating an initial average based on the first window of readings. This initial average serves as a baseline for further smoothing. A key component of the algorithm is the α factor, which controls the weighting between past and current averages. A higher α value assigns more importance to past data, while a lower α value makes the output more sensitive to recent changes. The algorithm iterates through the dataset using a sliding window, updating the current average for each window and applying exponential smoothing to the overall average. By combining data from the current and subsequent windows, the method effectively reduces noise and mitigates short-term fluctuations. This approach provides a stable and representative output while allowing gradual adaptation to change in the data. The flowchart in Figure 4 illustrates the overall process, enhancing the SMA approach by incorporating weighted factors that prioritize recent data.

The algorithm is divided into four primary steps: initialization, first window average calculation, alpha factor determination, and weighted moving average (WMA) computation. First, parameters such as window_size, size, and dataset readings [] are initialized. The variable sum is used to accumulate the data points within the window, while *i* serves as the loop index for processing.

In the next step, a loop iterates through the dataset, summing the data points in the first window while *i* is less than window_size and size. Once the loop completes, the first window’s average is calculated as avg = sum/window_size. The α factor is then determined to balance the contributions of the newly calculated average and the previous average. This weighting ensures that more emphasis is placed on recent data. Finally, the WMA is calculated by processing subsequent windows until the end of the dataset. This approach ensures that recent data are prioritized for analysis, while the influence of older data diminishes over time.

## 3 Experimental methods

### 3.1 Subjects

This study was approved by the Daegu University Institutional Review Board, and informed consent was obtained from all participants. The study group consisted of healthy individuals with no history of speech-related disorders, neurological or psychiatric conditions, and normal vision, hearing, and cognitive function as confirmed by the Korean Mini-Mental State Examination. All participants volunteered for the study and did not receive financial compensation for their participation. Personal information was securely managed in accordance with Daegu University Bioethics Committee guidelines (Approval No. 1040621-201907-HR-061-02). The data used for analysis were based on average values from five healthy male participants, with a mean age of 27.1 years (range: 24–38 years).

### 3.2 Exercise task

**FIGURE 5:**
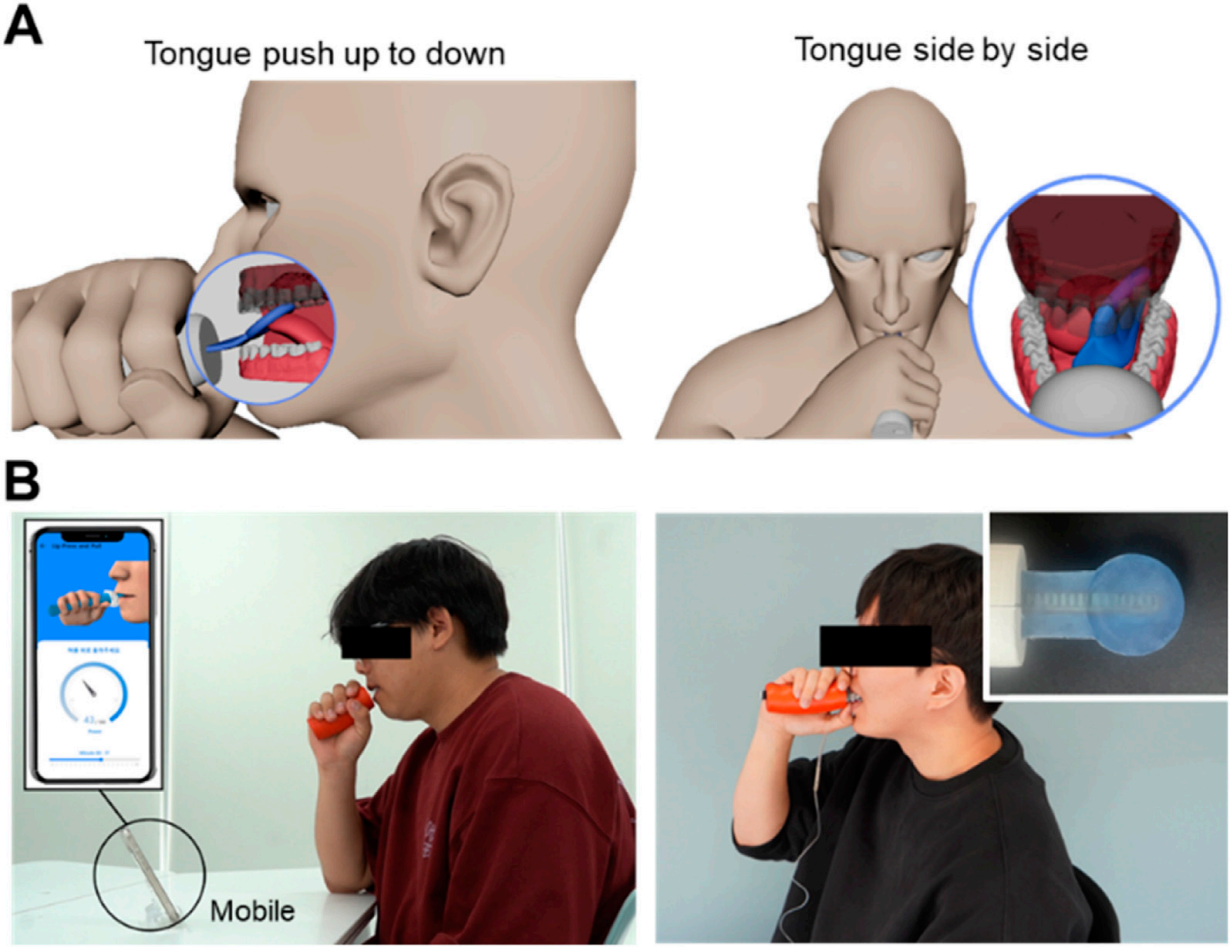
Schematic of tongue movements: **(A)** push up-to-down and side-to-side and **(B)** photograph of a participant wearing the manufactured device and performing an experiment.

The exercise method proposed in this study includes the tongue push-up (Figure 5A) and tongue side-to-side (Figure 5B) exercises. Together, these exercises form a comprehensive regimen to enhance oral motor function (Lin et al., 2021; Namiki et al., 2019). The tongue push-up exercise primarily targets tongue elevation, strengthening and improving the control of upward tongue movements essential for speech production and swallowing (Kamimura et al., 2019; Fukuoka et al., 2022). In contrast, the tongue side-to-side exercise focuses on lateral tongue movements (Lazarus et al., 2003; Coach and Owen, 2024), reinforcing the ability of the tongue to navigate side-to-side, which is vital for precise articulation and for producing lateralized speech sounds.

Regular practice of both exercises is expected to substantially improve overall tongue strength, enhance speech articulation clarity, and increase safety and efficiency in swallowing (Ibrahim et al., 2013; Arakawa et al., 2015; Mizuhashi and Koide, 2020; Clark et al., 2009; Health Care and Lowsky, 2013; Kappert et al., 2021). A photographic demonstration of the experiment using the implemented oral–motor exerciser is illustrated in Figure 5C. Before the experiment, participants rested for approximately 10 min and wore the device’s sensor to become familiar with its movements. The experiments were conducted in a quiet laboratory environment with background noise levels maintained between 30 and 35 dB SPL.

## 4 Results

### 4.1 Error rates according to window size and moving point

**FIGURE 6:**
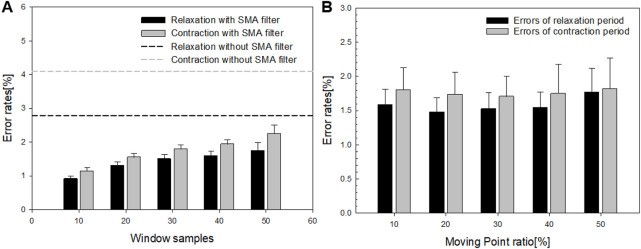
**(A)** Error rates corresponding to different window sizes and **(B)** the error rates relative to the moving point ratio.

First, the error rates were evaluated based on window size and moving point ratio to identify the optimal SMA filter parameters for the implemented oral–motor exerciser. Figure 6A presents the error rates for different window sizes, while Figure 6B details the error rates for varying moving point ratios. The dotted lines in Figure 6A represent the error rates without applying the SMA filter, which were measured at 4.14% for contraction movements and 2.85% for relaxation movements. When the window size was fixed at 10 samples, the error rates were minimized for both relaxation (black dotted lines) and contraction (gray dotted lines), reaching 0.91% and 1.13%, respectively. However, as the window size increased, the error rates rose, reaching approximately 2.2%. Similarly, as depicted in Figure 6B, the error rates for varying moving point ratios remained consistent, around 1.40%–1.45% for contraction movements and 1.7%–1.79% for relaxation movements. Additionally, we performed Pearson correlation analysis using IBM SPSS Statistics version 25.0 to assess the relationships between error rate, window sample size, and moving point. The results indicated a significant positive correlation between window sample size and error rate for both relaxation and contraction motions. In the case of relaxation Motion, a significant positive correlation of 0.977 (p < 0.05) was observed, suggesting that smaller window sizes are associated with lower error rates. In the contraction Motion, similarly, a significant positive correlation of 0.987 (p < 0.05) was noted. Given these findings, a window sample size of 10 was identified as the optimal parameter, considering the total sample length. Regarding the moving sample ratio, no significant differences in error rates were detected across both motions (relaxation; p > 0.05, contraction; p > 0.05). However, the lowest error rate (1.40%) occurred at a 20% moving sample ratio, a condition proposed in our experiment. Thus, this ratio was selected as the parameter for further application.

### 4.2 Bending angle characteristics of the fabricated oral-motor exerciser

**FIGURE 7:**
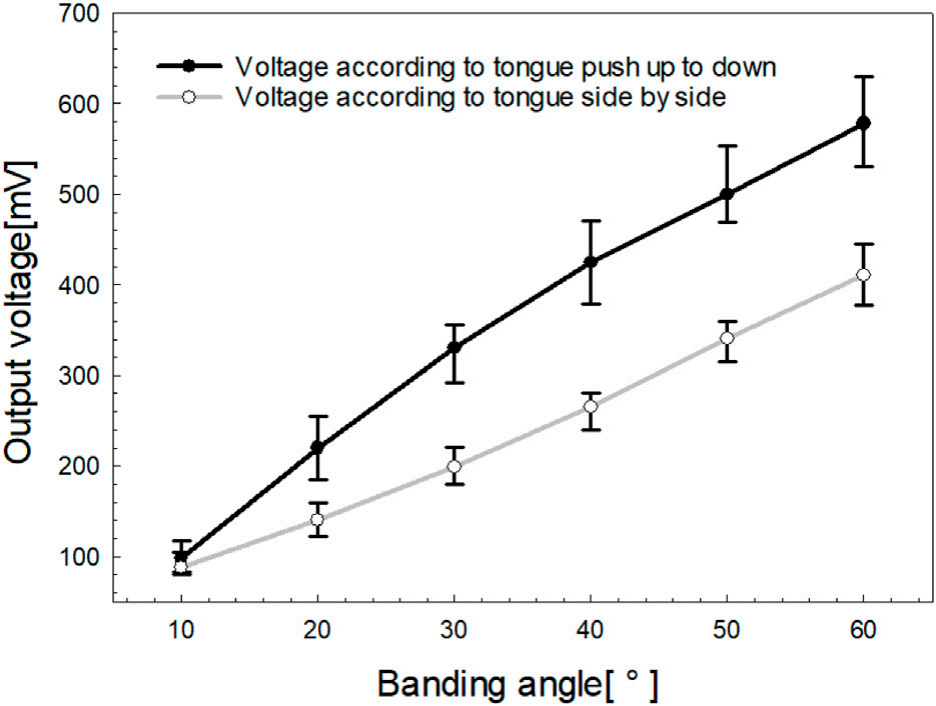
Output voltage characteristics are related to the tongue movement range.

Figure 7 illustrates the relationship between the bending angle and the output voltage of the sensor, capturing two types of motion: side-to-side and up-and-down. The output voltage was measured at 10° intervals, ranging from 10° to 60° for each motion. For the side-to-side motion, represented by the light gray line, the output voltage increased from 85 mV at 10° to 405 mV at 60°. In contrast, for up-and-down motion, represented by the black line, the output voltage increased linearly from 92 mV at 10° to 574 mV at 60°.

The greater voltage range observed in the up-and-down motion is attributed to the maximized resistance change caused by the bending trajectory. Meanwhile, the side-to-side motion resulted in a smaller voltage range owing to the twisting of the film, which limited the change in resistance. Although the voltage range differs between the two motions, the calibration function applied in this study normalizes individual variations in tongue movement, making the voltage range less critical. Instead, the key outcome is the linear relationship between output voltage and the tongue’s range of motion, as depicted in Figure 7.

**FIGURE 8:**
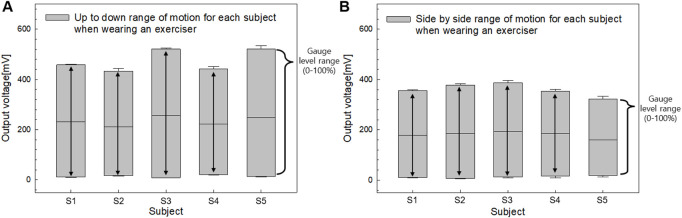
The **(A)** Output voltage characteristics for tongue push up-to-down and **(B)** side-to-side movements.

Figure 8 highlights the range of motion characteristics for the tongue movements of five participants, comparing side-to-side and up-and-down actions. Consistent with the findings in Figure 7, side-to-side movement exhibited approximately 25% lower output voltage compared to up-and-down motion, reflecting a smaller range of motion. Each participant displayed a unique range of motion, which was standardized to a gauge level using SMA filter-based calibration. Notably, Participants (S1 - S5) demonstrated a high range of motion for up-and-down movements but a comparatively low range for side-to-side movements. None of the participants exhibited a greater range of motion for side-to-side movements compared to those for up-and-down movements. Additionally, to determine if there were intra-group differences in the output voltages among bending angles for the two types of motion, we performed the Friedman Test, a non-parametric statistical analysis using IBM SPSS Statistics version 25.0. Post hoc comparisons were performed using Bonferroni’s method. As a result, both “side-to-side” and “up-and-down” indicated a statistically significant and steady increase in the output voltages across all bending angles (p < 0.001).

### 4.3 Relative pressure characteristics of the fabricated oral-motor exerciser

**FIGURE 9:**
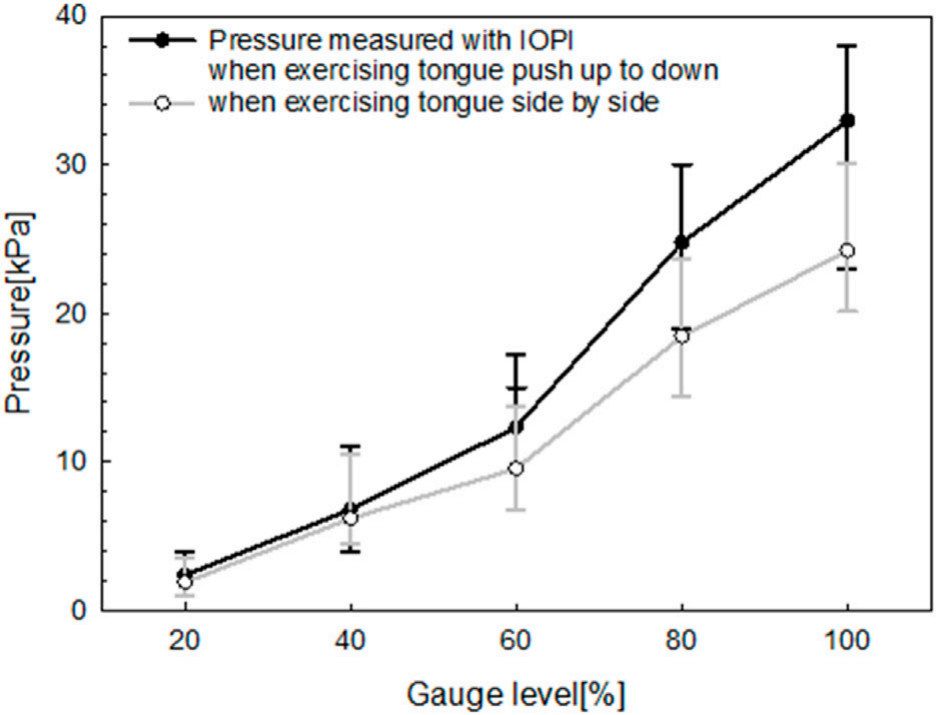
Relationship between gauge level and pressure measured simultaneously using the manufactured oral‒motor exerciser and IOPI.

To assess the pressure characteristics of the fabricated oral–motor exerciser under varying bending forces, an airflow tube from the IOPI was centrally attached to the device’s sensor. This setup allowed simultaneous measurement of the bending force, expressed as a gauge level, and the corresponding pressure (in kPa) generated by the user’s tongue movements. The gauge level index, representing bending strength, was recorded at five incremental levels: 20%, 40%, 60%, 80%, and 100%. This systematic measurement approach provided insights into the exerciser’s sensitivity and responsiveness to different levels of tongue exertion. Figure 9 illustrates the relationship between the gauge level index and the pressure measured simultaneously using the oral–motor exerciser and the IOPI. The results reveal a positive correlation between the gauge level and pressure for both tongue movement exercises: push up-to-down (black line) and side-to-side (gray line).

As the gauge level increased from 0% to 100%, both exercises demonstrated a progressive rise in pressure. However, the up-to-down movement consistently produced higher pressures than the side-to-side movement at all gauge levels. This distinction suggests that during the up-to-down motion, the tongue encounters greater resistance within the exerciser’s design, requiring it to exert more force. The linear progression of both curves indicates that the device reliably measures pressure across varying levels of tongue exertion. This feature provides valuable feedback for monitoring exercise intensity.

### 4.4 Characteristics according to the tongue movement trajectory

For pressure measurements, conventional air flow tube-type sensors require almost complete contact between the tongue and the palate. While this approach offers the advantage of providing absolute pressure measurements, thereby eliminating the need for individual user calibration, it poses challenges when analyzing pressure characteristics based on the tongue’s movement trajectory. To explore the relationship between pressure characteristics and tongue movement trajectories, this study used both the fabricated oral–motor exerciser and the IOPI. The performance of these devices was examined using an ultrasound imaging diagnostic system.

During the ultrasound measurements, participants gradually adjusted their tongue’s range of motion while performing an up-and-down pushing motion (Lin, 2014; Remijn et al., 2015; Chien et al., 2017). This motion was selected as the most suitable for this study owing to its compatibility with the ultrasound measurement range and the maximum trajectory of tongue motion, as opposed to the side-to-side movement.

**FIGURE 10:**
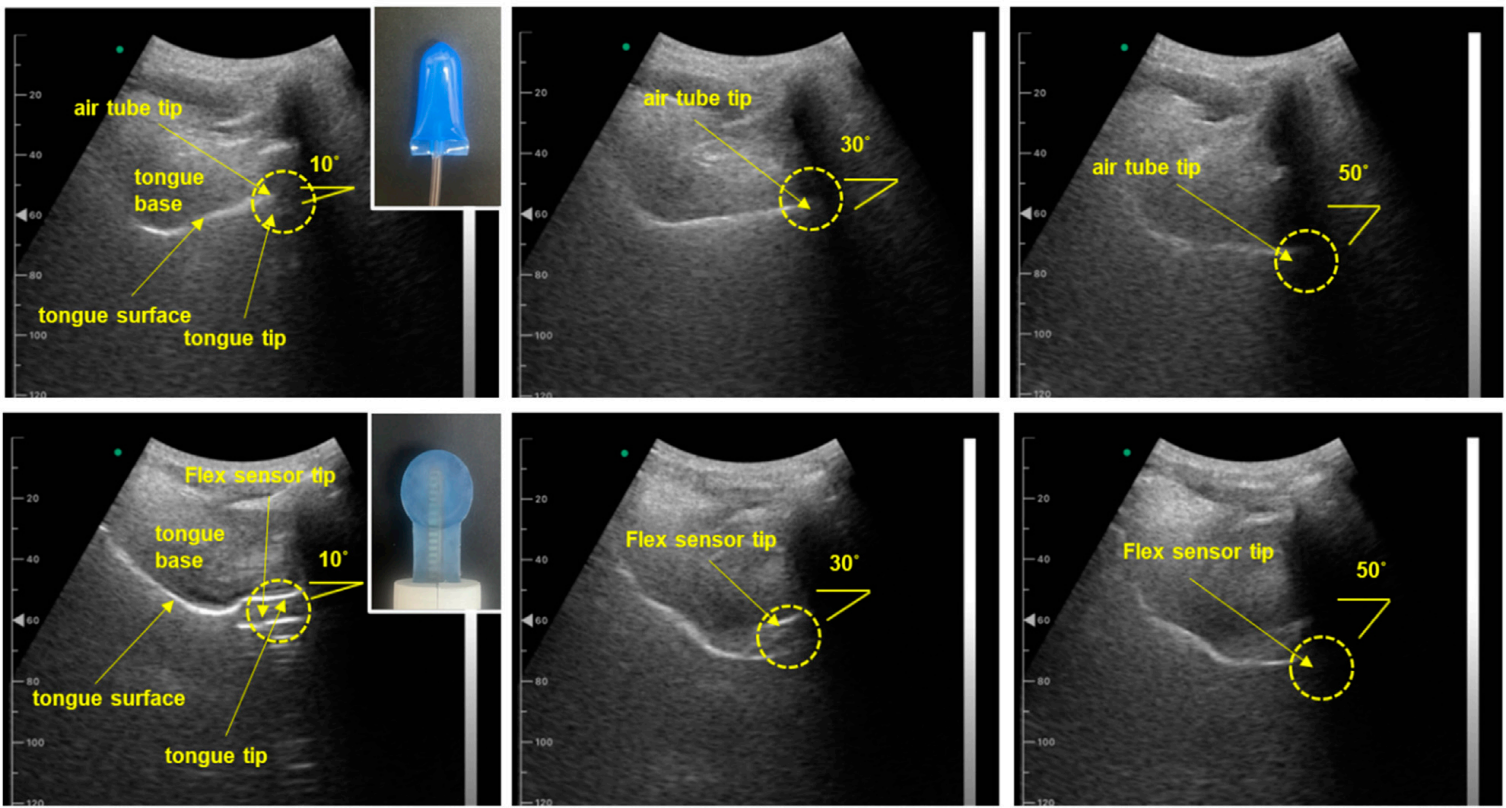
Sonography image for assessing tongue trajectory. (Top row) Subject one wearing the IOPI air flow tube sensor, with images captured at various tongue push-up angles ranging from 10° to 50°. (Bottom row) Subject one wearing the sensor from the proposed oral motor exerciser, with images captured as the participant progressively changed the range of tongue movement. (Participants wore both types of sensors, adjusting their tongue range progressively; angles were confirmed in real-time using sonography sagittal view images.)

Sonography provided a non-invasive, real-time method for assessing the tongue’s trajectory, enabling detailed analysis of its complex movements that are essential for functions like speech and swallowing (Abbott et al., 2020; Galén and Jost-Brinkmann, 2010; Manlises et al., 2020). During measurement, a convex ultrasound transducer was positioned under the participant’s chin to capture the sagittal plane of the tongue. This sagittal view provided a detailed longitudinal profile of the tongue, from the tip to the root. This setup allowed us to track the trajectories of the tongue’s surface, tip, dorsum, and root over time, generating a 2D trajectory map that visualized the dynamic patterns of tongue motion. The sonography imaging setup used for assessing the tongue’s trajectory is presented in Figure 10.

**FIGURE 11:**
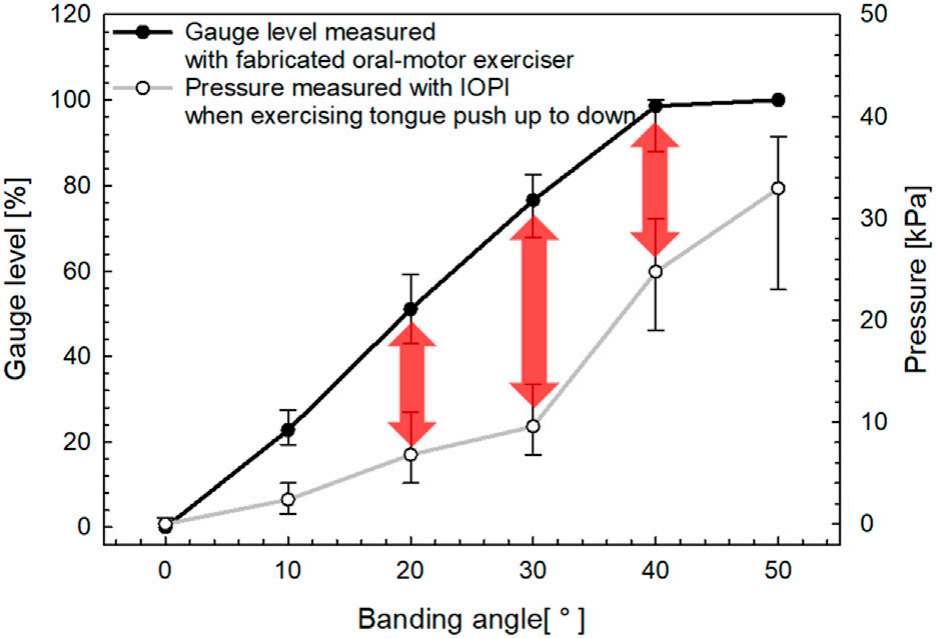
Sensor output characteristics based on tongue movement trajectory.

Furthermore, the sagittal view allowed us to confirm the IOPI’s airflow tube and the flexible sensor of the proposed device between the tongue and the palate as the tongue followed its movement trajectory. Figure 11 presents a graph illustrating the pressure and gauge levels measured across various movement trajectories.

The graph in Figure 11 presents a comparative analysis of gauge level measurements obtained from the fabricated oral–motor exerciser and pressure measurements derived from the IOPI during the up-and-down tongue motion. The x-axis represents the bending angle in degrees (°), while the left y-axis shows the gauge level as a percentage [%], and the right y-axis indicates the pressure in kilopascals [kPa].

The data in Figure 11 reveals a clear positive correlation between the bending angle and both measured parameters. The gauge level, represented by the solid black line, consistently increases with the bending angle, exhibiting a steeper incline compared to the pressure measurements recorded by the IOPI, depicted by the gray line. Notably, the pressure measured using the IOPI device shows a substantial rise beyond a bending angle of approximately 40°, as the tongue approaches the palate. This pattern underscores the difficulty in accurately evaluating the tongue’s range of motion for trajectories below 40°, as substantial pressure increases are only observed at higher angles.

## 5 Discussion

In this study, the proposed oral-motor exerciser ultimately aims to provide the user with immediate feedback on the characteristics of the tongue movement. Through experimental results and statistical approaches, we confirmed that the output voltage of the device according to the tongue bending angle showed a significant difference within the group for two types of exercises (push up-down, side by side) (p < 0.001, Friedman Test, a non-parametric statistical analysis). In other words, since the device sensitively reflects subtle differences in tongue bending angles in its output values, it can be effectively used to precisely measure delicate movements of the tongue. The while the oral–motor exerciser developed in this study shares several similarities with existing devices, such as the tonguometer, it incorporates a unique sensor component (Cunningham, 2020). The tonguometer, including models produced by companies like CranioRehab, is a sophisticated instrument designed to quantitatively assess tongue strength (Cheung et al., 2024). Primarily used in therapeutic settings, it measures the pressure exerted by the tongue, providing objective data on muscular performance. Typically, this device features a balloon-based sensor that detects the force exerted by the tongue and transmits the data to a display or application. Its immediate feedback mechanism enables users to monitor their exertion levels in real time during each trial.

Similarly, the IOPI, which was also used in this study, is a widely recognized oral–motor rehabilitation tool commonly employed in speech therapy, dysphagia management, and other rehabilitative practices (Oh, 2022; Adams et al., 2013; Pitts et al., 2022; Park et al., 2015). The IOPI features a small, air-filled pressure sensor that can be pressed with the tongue or lips. The exerted pressure is measured and displayed on a digital monitor, offering immediate, objective feedback on muscle strength. Both the IOPI and tonguometer share common features, namely, the use of air-filled balloon tubes and pressure sensors.

In contrast, the device developed in this study uses a resistance film as a sensor. This film provides a linear and stable output in response to bending motions and demonstrates high durability against temperature fluctuations and external pressure. By the results of Figures 10, 11, these characteristics allow the device to demonstrate more consistent performance and improved durability compared to conventional devices. Especially, the flexible sensor of the proposed oral–motor exerciser demonstrates a relatively linear increase in gauge level from approximately 10° to the maximum trajectory of 40°, indicating a broader and more gradual response across the range of motion. While reaching the maximum range of tongue motion is crucial, providing users with a real-time index of their range of motion is equally important. This feature enables users to receive immediate visual feedback on their motion range, enhancing the effectiveness of their exercise by promoting greater awareness and control.

### 5.1 Need for a novel sensor design for side-by-side tongue exercises

While the basic functionality for side-to-side tongue exercises has already been implemented, a critical challenge remains effectively and accurately detecting the direction of movement during these lateral exercises. Side-to-side tongue movement is essential for producing specific speech sounds, such as the “l” sound, and for enhancing overall tongue coordination. The objective is not to merely track the movement of the tongue from one side to the other but to precisely identify its position throughout the motion. While current devices, such as the IOPI and tongueometer, can measure vertical pressure, they fail to adequately capture the direction of lateral movements, a critical aspect of effective training and assessment.

To address this limitation, I propose designing a novel sensor capable of differentiating between left and right movements while providing clear, real-time feedback on direction. One potential solution involves using flexible resistive films or strain gauges that are sensitive enough to detect even subtle changes in the tongue’s direction. This design would enable us to monitor both the intensity and direction of tongue movements, which is critical for effective therapy.

Another crucial component is the mobile application, which should effectively visualize lateral tongue movements, allowing users to track their progress—how far they are moving to the left and right and whether their performance is improving over time. By establishing a Bluetooth connection with the proposed device, the app can enable seamless usage, allowing users to practice their exercises anywhere without being burdened by bulky equipment.

Successfully implementing direction detection for side-to-side tongue exercises could significantly benefit individuals struggling with speech clarity owing to poor lateral tongue control (Sanders and Mu, 2013; Mu and Sanders, 2010). Furthermore, considering that most individuals exhibit some degree of asymmetry in their facial muscles, targeted directional exercises could lead to noticeable improvements (van Alphen et al., 2017). This innovation would address a critical gap in current therapy options, offering a valuable tool for comprehensive oral–motor rehabilitation.

### 5.2 Limitations

This study has several limitations. First, for the universality of the proposed device, it is essential to standardize data across different age groups among healthy experimental participants. Subsequent prospective studies should evaluate its application to patient groups, such as those with dysarthria. Second, due to the small sample size of only five subjects, our study lacks the statistical power to perform variance analysis between groups. Thus, future studies should include a broader demographic to enable statistical approaches and interpretations that can validate the findings across various age groups and patient conditions. Third, our current device configuration is limited to side-by-side and push up-down tongue movements. To enhance its applicability in training, it is necessary to develop motion detection technologies for additional movements such as “bite-pull” and “tongue curls,” which are additionally utilized in rehabilitation exercise.

## 6 Conclusion

In this study, we developed a personalized oral–motor exerciser and a dedicated application for user feedback, enabling quantitative analysis of tongue push-up and lateral tongue exercises. Pressure changes corresponding to the device’s range of motion were also measured using the IOPI. The experimental results revealed a linear increase in pressure with bending strength and device range of motion, indicating that the proposed device can objectively assess a user’s tongue range of motion and exercise intensity. The device was specifically designed to quantify tongue movements and provide real-time visual feedback to users. By incorporating an SMA filter during calibration, the device accurately established individual movement ranges, enhancing training consistency. This approach addresses the limitations of conventional rehabilitation devices. Furthermore, this device has potential applications for patients with articulation or swallowing disorders. Future research should involve a broader range of participants, including individuals of different ages and medical conditions, to further validate the effectiveness of the proposed device.

## Data Availability

The original contributions presented in the study are included in the article/supplementary material, further inquiries can be directed to the corresponding author.
